# Oral Health: The First Step to Well-Being

**DOI:** 10.3390/medicina55100676

**Published:** 2019-10-07

**Authors:** Luca Fiorillo

**Affiliations:** 1 Department of Biomedical and Dental Sciences, Morphological and Functional Images, University of Messina, Policlinico G. Martino, Via Consolare Valeria, 98100 Messina, Italy; lucafiorillo@live.it; 2 Multidisciplinary Department of Medical-Surgical and Dental Specialties, Second University of Naples, 80100 Naples, Italy

**Keywords:** oral health, general health, well-being, epidemiology, interdisciplinary, multidisciplinary medicine

## Abstract

Scientific research in the medical field shows this constantly: health starts from the mouth. Having good oral health nowadays is not only aimed at tooth health, but as amply demonstrated in the literature, it is a starting point for the general health and well-being of our body. Retracing the latest scientific findings that demonstrate an interpolation between oral health, oral diseases, and systemic complications, literature support was brought to this manuscript. Oral health, as demonstrated, has potentially multi-organ systemic implications, and as the results of the recent literature demonstrate, these implications range from an insulin resistance, due to a periodontal disease, up to far more complex multi-organ systemic complications involving the cardiovascular system or even neurodegenerative pathology. Therefore, being able to improve oral health could have great systemic implications for the organism, for the prevention of pathologies, and therefore for society and for the quality of life in individuals.

Since the beginning, men with special skills, healing, and relief knowledge were highly sought after. These people were the first to deposit medical knowledge, which was handed down orally from generation to generation until it began to be written down. The first known written testimony of a dentist is the tomb of Hesy-Re, an Egyptian scribe who lived 4600 years ago and was described as “the best among those who treat teeth”. That Egypt was a land attentive to teeth is also witnessed by a papyrus with the first known recipe for toothpaste, a papyrus dating back more than 2000 years earlier, to the 4th century BC. More or less at that time, the Ancient Greeks, with Hippocrates (considered the “father” of medicine) and Aristotle, described [1,2,3]:

- the timing of the teething phases;
- cures for caries and inflamed gums;
- extractions;
- the use of metal wires to block moving teeth [4].

Medical wisdom, well attested and widespread throughout the Roman Empire, became increasingly rare with the end of the urban civilization that coincided with the beginning of the Middle Ages, while in the rest of the world, other civilizations flourished, like the Chinese, where in 700 AD the treatments were so advanced that in a medical text we talk about the use of a metallic amalgam for the treatment of caries. In Europe, with the revival of cities after the year 1000, the demand for dentists was once again felt, so much so that in 1210, the first Barbers’ guild was created in France, which had among its objectives the study and dissemination of methods of surgery, including those concerning the mouth [5].

In 1510 in Germany, shortly after the invention of movable type printing, the first book dedicated exclusively to dental care was published by Artzney Buchlein, with practical advice for barber-surgeons on drilling, prostheses, and extractions [6]. Just over 200 years later, in 1723, in full Enlightenment, the Frenchman Pierre Fauchard guaranteed himself the title of “Father of modern dentistry”, writing the book “Dental surgery”, the first text to describe a complete system for dental practice, from the functional anatomy of the various organs of the mouth to all the main operative care techniques [7].

The interest of medical research is focusing very much on the field of dentistry, which has been underestimated for years, as oral health, or oral pathology, could be the key to some systemic diseases. Many studies in the literature tend to analyze all the complications due to periodontopathy. Furthermore, there are important correlations of oral alterations associated with rare diseases. In fact, some authors report that some pathologies such as Von Willebrand disease, acrodermatitis enteropathica, Chediak–Higashi syndrome, and others have important correlations with oral health [8,9,10,11].

In recent decades, numerous clinical evidences have highlighted an association between dental disorders and cardiovascular diseases and diabetes, lung diseases, and obstetric complications. Periodontal diseases could therefore have serious systemic effects through blood-borne dissemination of pathogenic bacteria and also through the negative inflammation role (Figure A1) [12]. Periodontal disease negatively affects the whole body, and it has a close correlation with diabetes and other systemic diseases mentioned above. Periodontitis could also be an initial sign of diabetic pathology since the prevalence of periodontitis in diabetic subjects is double or triple compared to non-diabetic subjects [13]. Controlling diabetes is complicated for patients when they suffer periodontal disease. Patients suffering from periodontitis and diabetes simultaneously are exposed to developing complications of cardiovascular, renal, and retinopathic diseases. Recently, the presence of some bacteria responsible for periodontal disease has been reported in the brain tissue of patients suffering from Alzheimer’s disease [14,15].

Surely, in-depth research in medicine, biology, and oral pathology will, in addition to the prevention of some systemic diseases, lead to the early diagnosis of other diseases in the near future, including rare diseases.

According to some epidemiological data, dental disease is concentrated among populations with low socioeconomic status [16]. Dental care is not publicly funded, and many people in the Western world must therefore make difficult financial choices when accessing dental care, this without considering patients from developing countries. Families who live in poverty have difficulty meeting their basic needs as they can be unaffordable. Many families are unable to afford both a nutritious diet and dental care [17]. This is disturbing given the links between a healthy diet and both overall health and dental health. Considering that economic difficulties can negatively affect the quality of oral health, and in turn that bad oral health can negatively affect general health, it is possible to deduce that the socio-economic conditions of a citizen have a short-term influence on solvable oral pathologies that, in the long term, besides becoming irreversible chronic oral diseases, also cause a decay in the general health of the individual (Figure A2) [18,19,20]. Among the most demanding treatments from an economic point of view, we must certainly mention implant-prosthetic rehabilitation treatments; it is also important to remember that there is a strong psychological component associated with patients’ fear of dental care [21,22].

Furthermore, by encouraging scientific research in the interdisciplinary field of dentistry, it would be possible to obtain useful results for the resolution and prevention of systemic diseases, as already mentioned. This would help to inform clinicians, dentists, who would be able to intercept and resolve pathologies that would be very expensive for the public health system.

## Figures and Tables

**Figure A1:**
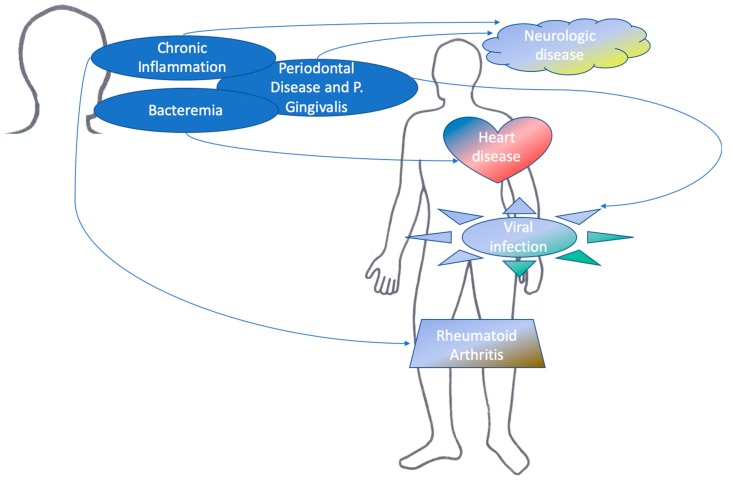
Example of how oral health can affect systemic health.

**Figure A2:**
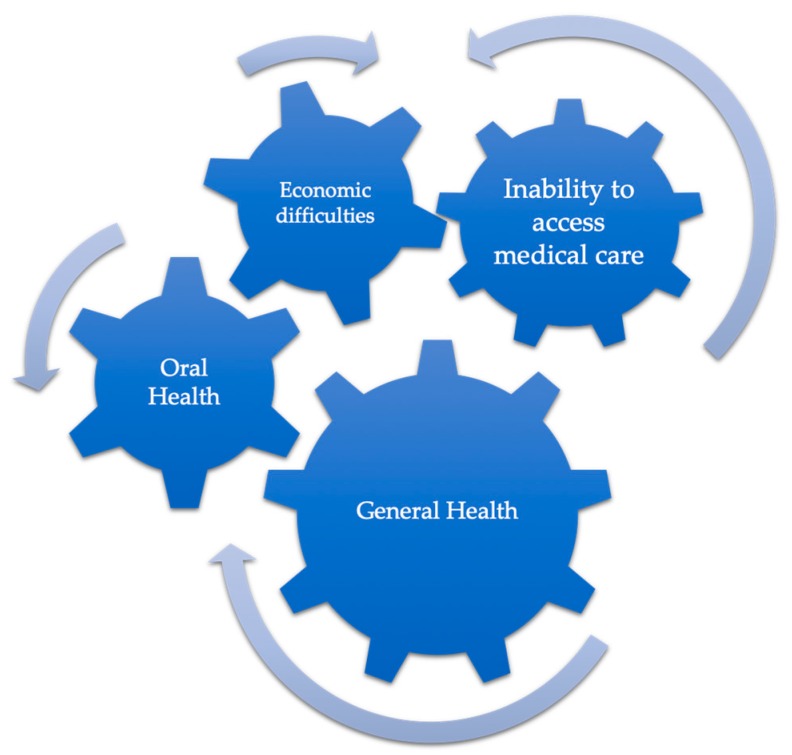
Brief outline of how economic social situation can influence the general health of the organism, also through oral health.

## References

[1] Leclant J. (1987). Medicine and dentistry in ancient Egypt. Bull. Acad. Natl. Chir. Dent. (1983).

[2] Blackburn S.P. (1977). A short history of dentistry in ancient times. CAL Certif. Akers Lab..

[3] Yapijakis C. (2009). Hippocrates of Kos, the father of clinical medicine, and Asclepiades of Bithynia, the father of molecular medicine. Review. In Vivo (Athens Greece).

[4] El-Assal G.S. (1972). Ancient Egyptian medicine. Lancet.

[5] Bifulco M., Amato M., Gangemi G., Marasco M., Caggiano M., Amato A., Pisanti S. (2016). Dental care and dentistry practice in the Medieval Medical School of Salerno. Br. Dent. J..

[6] Sigron G. (1984). Dentistry in the early Middle Ages. Schweiz. Monatsschrift Zahnmed. Rev. Mens. Suisse Odontol. Stomatol. Riv. Mens. Svizzera Odontol. Stomatol..

[7] Lynch C.D., O’Sullivan V.R., McGillycuddy C.T. (2006). Pierre Fauchard: The ‘father of modern dentistry’. Br. Dent. J..

[8] Hanisch M., Hoffmann T., Bohner L., Hanisch L., Benz K., Kleinheinz J., Jackowski J. (2019). Rare Diseases with Periodontal Manifestations. Int. J. Environ. Res. Public Health.

[9] Fiorillo L., De Stefano R., Cervino G., Crimi S., Bianchi A., Campagna P., Herford A.S., Laino L., Cicciù M. (2019). Oral and Psychological Alterations in Haemophiliac Patients. Biomedicines.

[10] Žaliūnienė R., Aleksejūnienė J., Brukienė V., Pečiulienė V. (2015). Do hemophiliacs have a higher risk for dental caries than the general population?. Medicina.

[11] Ravi D.K., Taylor W.R., Singh N.B., Poston B., Mickel C., Coco M. (2018). The “Journal of Functional Morphology and Kinesiology” Journal Club Series: Highlights on Recent Papers in Motor Control and Learning. J. Funct. Morphol. Kinesiol..

[12] Poole D.F., Newman H.N. (1971). Dental plaque and oral health. Nature.

[13] Cervino G., Terranova A., Briguglio F., De Stefano R., Famà F., D’Amico C., Amoroso G., Marino S., Gorassini F., Mastroieni R. (2019). Diabetes: Oral health related quality of life and oral alterations. BioMed Res. Int..

[14] Dominy S.S., Lynch C., Ermini F., Benedyk M., Marczyk A., Konradi A., Nguyen M., Haditsch U., Raha D., Griffin C. (2019). Porphyromonas gingivalis in Alzheimer’s disease brains: Evidence for disease causation and treatment with small-molecule inhibitors. Sci. Adv..

[15] Cicciù M. (2016). Neurodegenerative disorders and periodontal disease: Is there a logical connection?. Neuroepidemiology.

[16] Manski R.J., Moeller J.F., Chen H., Schimmel J., St Clair P.A., Pepper J.V. (2014). Dental usage under changing economic conditions. J. Public Health Dent..

[17] Trovato F.M., Martines G.F., Brischetto D., Catalano D., Musumeci G., Trovato G.M. (2016). Fatty liver disease and lifestyle in youngsters: Diet, food intake frequency, exercise, sleep shortage and fashion. Liver Int..

[18] Snow P., McNally M.E. (2010). Examining the implications of dental treatment costs for low-income families. J. Can. Dent. Assoc..

[19] Žemaitienė M., Grigalauskienė R., Vasiliauskienė I., Saldūnaitė K., Razmienė J., Slabšinskienė E. (2016). Prevalence and severity of dental caries among 18-year-old Lithuanian adolescents. Medicina.

[20] Kalėdienė R., Starkuvienė S., Petrauskienė J. (2008). Inequalities in life expectancy by education and socioeconomic transition in Lithuania. Medicina.

[21] Genc T., Duruel O., Kutlu H.B., Dursun E., Karabulut E., Tozum T.F. (2018). Evaluation of anatomical structures and variations in the maxilla and the mandible before dental implant treatment. Dent. Med. Probl..

[22] Bruno A., Muscatello M.R.A., Pandolfo G., Ciura G., Quattrone D., Scimeca G., Mento C., Zoccali R.A. (2018). Does Personality Matter? Temperament and Character Dimensions in Panic Subtypes. Arch. Neuropsychiatry.

